# Silicon applications in rice plants alter the stylet probing behaviors of *Glyphepomis spinosa* (Hemiptera: Pentatomidae)

**DOI:** 10.1093/jisesa/ieae046

**Published:** 2024-04-10

**Authors:** Walter Baida Garcia Coutinho, Franciele Cristina da Silva, José Alexandre Freitas Barrigossi, André Cirilo de Sousa Almeida, Flávio Gonçalves de Jesus

**Affiliations:** Federal Goiano Institute—Campus Urutaí, Rodovia Geraldo Silva Nascimento, Km 2,5, Urutaí, 75790-000 Goiás, Brazil; Federal Goiano Institute—Campus Urutaí, Rodovia Geraldo Silva Nascimento, Km 2,5, Urutaí, 75790-000 Goiás, Brazil; Embrapa Rice and Beans, Rodovia GO 462, Km 12, Santo Antônio de Goiás, 75375-000 Goiás, Brazil; Federal Goiano Institute—Campus Urutaí, Rodovia Geraldo Silva Nascimento, Km 2,5, Urutaí, 75790-000 Goiás, Brazil; Federal Goiano Institute—Campus Urutaí, Rodovia Geraldo Silva Nascimento, Km 2,5, Urutaí, 75790-000 Goiás, Brazil

**Keywords:** EPG, induced plant resistance, *Oryza sativa*, silicon amendment

## Abstract

The stink bug *Glyphepomis spinosa* Campos & Grazia (Hemiptera: Pentatomidae) is a potential rice pest in Brazil. This study evaluates the interaction between silicon sources and 3 rice cultivars (BRS Esmeralda, Canela de Ferro, and IRGA 417) and examines how increasing silicon levels affect the stylet probing behavior of *G. spinosa*. The experiment was set up in a completely randomized design with a 3 × 3 factorial scheme (silicon sources: calcium silicate, potassium silicate, a control, and 3 rice cultivars). Fertilizing rice plants with Si altered the probing behavior of the stink bug *G. spinosa*. The cultivar interaction by Si source was significant in a few variables. This was evidenced by longer periods without ingestion, prolonged time to the first stylet probe (initial probing), and less time spent in cellular maceration. This result supports the use of electropenetrography as a tool to evaluate resistance inducers in plants.

## Introduction

Pentatomid stink bugs are important pests for rice crops (*Oryza sativa* L.: Poaceae) throughout the world (Bhavanam et al. 2021). Stink bug damage in rice begins at the seedling stage, causing death of the central portion of the plant stem (dead heart) and continues to the reproductive stage, causing white panicles and grain damage (Krinski and Foerster 2017, Almeida et al. 2020a). These pests can reduce yields by as much as 80%, depending on the species, and represent one of the most significant insect problems affecting global rice production (Barrigossi and Martins 2015).

Several pentatomid species can be found in Brazilian rice crops, whether in irrigated, flooded, or upland cultivation systems. The most common species are the rice-stalk stink bug, *Tibraca limbativentris* Stål, the small rice stink bug, *Oebalus poecilus* (Dallas), and *Oebalus ypsilongriseus* (De Geer) (Hemiptera: Pentatomidae) (Krinski and Foerster 2017, Almeida et al. 2020a). In Goiás and Tocantins state, populations of *Glyphepomis spinosa* Campos & Grazia (Hemiptera: Pentatomidae) injury rice plants, with injury symptoms similar to those of *T. limbativentris* (Alves et al. 2012, Almeida et al. 2020a, 2020b). *Glyphepomis spinosa* damage can either occur during the vegetative phase with symptoms of dead heart or during the reproductive phase, resulting in white panicles or empty grains (Silva 2022).

Pentatomid pests in rice crops are usually controlled by chemical insecticides. Neonicotinoids, pyrethroids, and acephate are used most often, and no phytosanitary products have been registered for *G. spinosa* (AGROFIT 2021). This situation increases the risks to environmental and human health, selection of resistant insects, adverse effects on natural enemies, and higher production costs resulting from repeat applications (Krinski and Foerster 2016, Guedes 2017, Woodcock et al. 2017, Stehle et al. 2018). Given the threat that these species pose to rice production, management practices that incorporate different tactics that provide longer protection and ease of use need to be developed (Reagan and Megan 2019). Resistance induced by silicon (Si) amendments are promising tools for managing stink bugs in rice crops (França et al. 2018).

Rice plants are typical Si accumulators among grass species (Ma et al. 2006). Si confers plant resistance via depositions in cell walls that form physical barriers and hinder probing by sucking insects (Yang et al. 2018) or reduce the digestion efficiency of herbivorous insects (Kvedaras et al. 2010). This element can also elicit plant resistance induction processes (Reynolds et al. 2016) and influence indirect plant defenses by releasing volatile compounds and attracting natural enemies (Sidhu et al. 2013). Several studies have demonstrated the beneficial effects of Si on inducing resistance in rice plants. For example, Franca et al. (2018) showed that greater accumulation of Si in the BRS Esmeralda rice cultivar decreased damage caused by *T. limbativentris*. Yang et al. (2018) found that the probing of *Nilaparvata lugens* (Stål) (Hemiptera: Delphacidae) was lower in rice plants fertilized with Si. Sidhu et al. (2013) and Pelosi et al. (2022) showed that soil correction with Si helped manage *Diatraea saccharalis* (Fabricius) (Lepidoptera: Crambidae) by reducing the damage caused by insect probing.

The stylet probing (or penetration) behavior of sucking insects in plants with Si-mediated resistance needs to be researched. Understanding stylet penetration can help identify resistance factors or mechanisms in rice plants (Yang et al. 2018). In electropenetrography (EPG), the insect and plant are made part of the same electrical signal in order to record voltage changes that represent otherwise invisible stylet penetration behaviors inside the plant. It allows observation and quantification of behaviors otherwise hidden in the opaque food substrate, can be used to improve our understanding of the feeding process of piercing-sucking insects, and can assist in the selection of insect-resistant plants (Yang et al. 2017, Almeida et al. 2020b, Lucini et al. 2021).

The objective of the current study was to determine whether plants fertilized with silicon affect the probing behavior of *G. spinosa*. This information may provide additional evidence that Si induces resistance to stink bugs in rice plants and will improve our understanding of the probing behavior of stink bugs in plants fertilized with Si.

## Materials and Methods

### Location and Experimental Design

All experiments were carried out at the Integrated Pest Management Laboratory of the Federal Goiano Institute—Campus Urutaí, Urutaí, Goiás, Brazil. A completely randomized design was used with a factorial scheme (3 × 3), consisting of 9 treatments (2 silicon sources + control × 3 rice cultivars). The rice cultivars BRS Esmeralda (moderately resistant cultivar; Genealogy: CNAx4909-68-M-M2-PY2 x BRS Primavera), Canela de Ferro (resistant cultivar; Genealogy: Brazil, breeding line), and IRGA 417 (susceptible cultivar; Genealogy: New Rex/IR19743-25-2-2 x BR IRGA 409) were selected because they possess different levels of resistance to *T. limbativentris*, which has a similar probing behavior to that of *G. spinosa* (Barrigossi and Martins 2015, Almeida et al. 2021). The Si sources were: 1—potassium silicate—K_2_SiO_3_ 1% (12% SiO_2_ and 12% K_2_O) sprayed on plants until runoff (20 mL/plant) at 15, 30, and 45 days after planting (DAP), 2—Ca silicate—wollastonite (CaSiO_3_) (48.2% CaO and 51.7% SiO_2_) applied to the soil (2.5 g per pot or 600 kg ha^−1^), and 3—control (without silicon applications).

### Stink Bug Rearing

Adults of *G. spinosa* were obtained from Embrapa Rice and Beans (Santo Antônio de Goiás, Goiás, Brazil), where mass rearing was maintained according to the methodology described by Alves et al. (2012). The adults were kept on the susceptible cultivar BR IRGA 409, which was grown in plastic pots (8 L) with a substrate (3:1:1–3: soil (red dystrophic oxisol), 1: sand and 1: organic compounds (cattle manure)). Soil correction and fertilization were carried out according to the recommendations for rice crops in the Cerrado ecoregion of Brazil (Sousa and Lobato 2004) and the plants were irrigated as needed. The plants and insects were placed in a greenhouse (LD 12:12 h, 25 ± 8 °C, 65 ± 20% RH) for female oviposition. After oviposition, the eggs were collected (using scissors to cut a fragment of the leaf with the eggs) and transferred to plastic containers (11 × 11 × 3.5 cm) lined with moistened paper towels and kept in the laboratory (*T* 25 ± 2 °C, RH 70 ± 10%, 14-h photoperiod) until hatching. The nymphs were kept under the same conditions until reaching the second instar when they were transferred to rice plants (BR IRGA 409, covered with voile mesh cages (15-cm Ø and 50-cm high) and maintained until reaching the adult stage).

### Cultivar Sowing

Seeds of the cultivars were sown in 5-L pots (5 plants per pot) containing the same substrate and kept under the same conditions described above. At 50 DAP, the rice plants were separated and taken to the laboratory to be used once in the EPG recordings.

### Probing Behavior—Electropenetrography

EPG data were collected using a 4-channel, AC–DC monitor (Backus and Bennett 2009) (EPG Technologies, Inc., Gainesville, FL) that was connected to a computer for obtaining and storing the data via the EPG System program (detailed below).

Adult *G. spinosa* females (12–15 days old) were used to analyze probing behavior. These insects were fasted for 5 h before the bioassay (without water) and without an acclimatization period for the tested food sources. Afterward, the insects were immobilized in a Petri dish with adhesive tape, and the lipid layer of the insect pronotum was removed with dental sandpaper (Lucini and Panizzi 2016). The insects were wired by gluing one end of a gold wire (0.127 mm in diameter and 3.5 cm in length) (Sigma Aldrich, Barueri, São Paulo, Brazil) to a copper wire (3 cm in length) that had been soldered to a brass nail. A small loop was made at the other end of the gold wire to increase the contact area with the insect and improve electrical conductivity (Cervantes et al. 2018). The gold wire was attached to the surface of the insect using a silver glue made with 10 μm silver flake (Sigma Aldrich, Barueri, SP, Brazil), water, silver flakes, and white glue (Cascorez, Jundiai, SP, Brazil) (1:1:1 wt/vol/vol) (Cervantes et al. 2018). The glue was then allowed to dry for approximately 40 min before recording. Each stink bug was EPG-recorded for 16 h under laboratory conditions (25 ± 2 °C) and constant light (Backus et al. 2007). A total of 15 subjects were recorded (successfully) per treatment in a completely randomized design.

The recordings were made by using an input impedance of 10^7^ Ohms for each of the 4 channels, a voltage of 50 mV alternating current (AC) via the plant electrode, and a gain set to 400 × (actual gain 4,000 with 10× multiplier) (Almeida et al. 2020b). An offset control was used to avoid rectifier fold-over and retain native waveform polarity after rectification (Backus and Bennett 2009). Voltage outputs were amplified and captured at a rate of 100 Hz per channel, using WinDaq DI-710 equipment (Dataq Instruments, Akron, OH) and recorded on a computer with WinDaq Lite software (Dataq Instruments, Akron, OH). The amplifiers, plants, and insects were kept inside a Faraday cage during the recordings to reduce external electrical noise.

Waveforms were identified and named according to the nomenclature proposed by Almeida et al. (2020b), who studied the probing behavior of *Tibraca limbativentris* on rice plants. Pentatomid waveforms have been intensively studied, with a comparison being made between 8 species of stink bugs on different hosts. In general, the waveforms are similar (Panizzi et al. 2021), which allows the waveforms to be identified based on EPG work with other pentatomid species, rather than doing the preliminary histology. A brief description of the recorded waves and their biological significance is summarized in Table 1. Five behaviors represented by waveforms were assessed using the variables described below: non-probing, pathway, xylem ingestion, cell rupture, and short ingestion of macerated tissues. Five variables were evaluated: time until first probe from start of EPG (t_1Pr or TmFrstPrbFrmStrt), waveform duration per insect (WDI), number of waveform events per insect (NWEI), waveform duration per event per insect (WDEI), and percentage of recording time spent in probing (PRTP) (Backus et al. 2007, Sarria et al. 2009, Ebert et al. 2015, Cervantes et al. 2016).

**Table 1.** Waveforms recorded using the electropenetrography (EPG) *Glyphepomis spinosa* fed on stalks of different rice cultivars treated with different sources of silicon, and their biological significance

Phase	Family	Type/subtype	*G. spinosa* biological meaning
Non-probing	–	Z	Insect immobile on plant surface
		Np	Walking on plant surface
Pathway	P	Tl1	Penetration of stylets and salivary sheath secretion
Ingestion	I	Tl2	Xylem sap ingestion
Salivation	I	Tl3a	Cellular laceration and enzymatic maceration of stalk tissues
Ingestion	I	Tl3b	Short ingestion of macerated tissues

Source: Almeida et al. 2020b.

### Statistical Analysis

The data were tested for residual normality (Shapiro–Wilk test) and homogeneity of variance (Bartlett test). The EPG data were entered in the INFEST Insect Feeding Behavior Statistics software (available at https://arsilva.shinyapps.io/infest/), which provided spreadsheets. Next, the data were tested for their suitability for different generalized linear models (Hnp Package). The t_1Pr variable was fit to a model based on the negative binomial model (Mass Package). Afterward, the factor levels were distinguished (Sqldf Package) for multiple comparisons of means (Multcomp Package) and summarization of means (Rmisc Package). The semiparametric analysis method was adopted for the remaining variables. Thereafter, the data were submitted to Fisher’s LSD test for multiple comparisons of means (ExpDes Package). All analyses were performed in the R statistical computing environment version 4.0.2.

## Results

### Probing Behavior—Electropenetrography

Silicon influenced the probing behavior of *G. spinosa* on different rice cultivars. Time until the first probe (t_1Pr) differed significantly among cultivars and Si sources (Table 2). Also, the interaction cultivar by Si source was significant. Esmeralda and IRGA 417 fertilized with potassium silicate, and Canela de Ferro and BRS Esmeralda fertilized with calcium silicate, stood out in this regard, producing the longest time until the first probe.

**Table 2.** Time spent to first probe (t_1Pr) in rice cultivars fertilized with different Si sources after infestation of *Glyphepomis spinosa*

Cultivar	t_1Pr—TmFrstPrbFrmStrt (min)		
	Control	K_2_SiO_3_	CaSiO_3_
Canela de Ferro	5.05 ± 1.18 bB	6.54 ± 1.86 bB	22.63 ± 6.48 aA
BRS Esmeralda	11.34 ± 3.39 abB	23.56 ± 7.82 aA	33.48 ± 10.38 aA
IRGA 417	27.40 ± 8.72 aA	36.28 ± 13.93 aA	6.78 ± 1.38 bB
Cultivar	*P* < 0.001	Deviance_2,126_ =140.7	
Si sources	*P* < 0.001	Deviance_2,126_ = 136.2	
Cultivar × Si sources	*P* < 0.001	Deviance_4,126_ = 104.5	

Means followed by the same lower-case letter within a column and upper-case letter within a row do not differ (LSD test, *P* ~ 0.05).

Table 3 shows the results of WDI. Adult *G. spinosa* spent longer in non-probing activities (Z and Np waveforms) on the surface of rice plants than in probing activities (Tl1, Tl2, Tl3a, and Tl3b waveforms). Overall non-probing duration was significantly longer in cultivars IRGA 417 and BRS Esmeralda fertilized with calcium silicate and potassium silicate (Table 3). The Si source and cultivar did not influence the overall pathway durations (Tl1 wave); also, the interaction was not significant. The Si source and the cultivar by Si source interaction did not influence the overall duration of xylem ingestion (waveform Tl2); however, adult *G. spinosa* was ingested for a longer time on the xylem of IRGA 417. Rice cultivars were influenced by the source of Si and cultivar regarding overall laceration and maceration of stalk tissue (wave Tl3a). Adult *G. spinosa* spent less time on this activity in BRS Esmeralda and IRGA 417 fertilized with calcium silicate and potassium silicate. Adult *G. spinosa* also spent less time in the short periods of ingesting macerated tissues (wave Tl3b) on BRS Esmeralda and IRGA 417 fertilized with calcium silicate. Si source and the cultivar by Si source interaction were not significant.

**Table 3.** Waveform duration per insect *Glyphepomis spinosa* (WDI—min) on different rice cultivars with different Si sources

Waveform	Cultivar	WDI—Waveform duration per insect		
		Control	K_2_SiO_3_	CaSiO_3_
Z + Np	Canela de Ferro	534.3 ± 88.39 bB	767.7 ± 37.48 bA	622.5 ± 85.87 bAB
	BRS Esmeralda	571.1 ± 95.07 bB	819.1 ± 66.29 aA	815.0 ± 66.66 aA
	IRGA 417	826.9 ± 24.74 aB	863.8 ± 18.89 aA	858.7 ± 26.03 aA
	Cultivar	*P* < 0.001	*F*_2,126_ = 8.13	
	Si sources	*P* = 0.004	*F*_2,126_ = 5.81	
	Cultivar × Si sources	*P* = 0.257	*F*_4,126_ = 1.35	
Tl1	Canela de Ferro	5.66 ± 1.54 aA	2.71 ± 0.48 aA	9.58 ± 4.78 aA
	BRS Esmeralda	19.86 ± 11.60 aA	1.48 ± 0.16 aA	7.59 ± 4.49 aA
	IRGA 417	5.36 ± 1.35 aA	2.44 ± 0.49 aA	14.58 ± 10.47 aA
	Cultivar	0.745	*F*_2,126_ = 0.29	
	Si sources	0.134	*F*_2,126_ = 2.06	
	Cultivar × Si sources	0.348	*F*_4,126_ = 1.12	
Tl2	Canela de Ferro	12.61 ± 7.07 abA	5.84 ± 2.16 bA	1.09 ± 0.79 cA
	BRS Esmeralda	7.36 ± 2.98 bA	6.96 ± 3.80 bA	6.16 ± 3.05 bA
	IRGA 417	22.39 ± 9.17 aA	10.3 ± 3.46 aA	12.92 ± 3.48 aA
	Cultivar	0.040	*F*_2,126_ = 3.33	
	Si sources	0.115	*F*_2,126_ = 2.21	
	Cultivar × Si sources	0.668	*F*_2,126_ = 0.59	
Tl3a	Canela de Ferro	308.76 ± 82.40 aA	115.25 ± 27.37 aB	221.56 ± 70.63 aAB
	BRS Esmeralda	296.44 ± 79.08 aA	76.32 ± 46.59 bB	105.36 ± 61.66 abB
	IRGA 417	79.44 ± 23.14 bA	56.37 ± 15.82 bB	55.85 ± 17.50 bB
	Cultivar	0.003	*F*_2,126_ = 6.11	
	Si sources	0.004	*F*_2,126_ = 5.80	
	Cultivar × Si sources	0.294	*F*_4,126_ = 1.25	
Tl3b	Canela de Ferro	77.97 ± 39.55 aA	68.39 ± 14.21 aA	105.18 ± 26.27 aA
	BRS Esmeralda	59.41 ± 24.57 abA	56.11 ± 21.17 aA	26.09 ± 5.92 bA
	IRGA 417	25.81 ± 4.05 bA	27.00 ± 5.72 bA	17.91 ± 3.84 bA
		0.18	0.32	0.004
	Cultivar	0.001	*F*_2,126_ = 6.90	
	Si sources	0.954	*F*_2,126_ = 0.46	
	Cultivar × Si sources	0.478	*F*_4,126_ = 0.88	

Means followed by the same lower-case letter within a column (same variable) and upper-case letter within a row do not differ (LSD test, *P* ~ 0.05). Z + Np, non-probing activities; Tl1, penetration of stylets; Tl2, xylem sap ingestion; Tl3a, cellular maceration; Tl3b, ingestion of macerated tissue.

Rice cultivar interaction by Si source and Si source both significantly influenced the NWEI of *G. spinosa* adults, as shown in Table 4. Behaviors significantly affected were laceration and maceration in plant stem tissue (Tl3a), and the short periods of ingestion of macerated tissues (wave Tl3b). The number of Tl3a events was higher in plants fertilized with Si in the Canela de Ferro and BRS Esmeralda cultivars compared to the control (no Si); however, the opposite happened in the IRGA 417 in which Tl3a events were more frequent in plants not treated with Si. A similar result was observed for Tl3b; ingestion events occurred more frequently in Canela de Ferro with calcium silicate, whereas for IRGA 417 this pattern was reversed.

**Table 4.** Number of waveform events for *Glyphepomis spinosa* (NWEI) probing on different rice cultivars fertilized with different Si sources

Waveform	Cultivar	NWEI—Number of waveform events per insect		
		Control	K_2_SiO_3_	CaSiO_3_
Z + Np	Canela de Ferro	5.9 ± 1.07 aA	9.2 ± 1.52 aA	8.4 ± 2.03 aA
	BRS Esmeralda	7.9 ± 2.75 aA	5.6 ± 0.52 aA	8.0 ± 3.08 aA
	IRGA 417	7.4 ± 0.88 aA	6.7 ± 1.09 aA	6.2 ± 0.57 aA
	Cultivar	*P* = 0.751	*F*_2,126_ = 0.28	
	Si sources	*P* = 0.942	*F*_2,126_ = 0.59	
	Cultivar × Si sources	*P* = 0.514	*F*_4,126_ = 0.82	
Tl1	Canela de Ferro	5.3 ± 1.11 aA	8.3 ± 1.58 aA	6.8 ± 1.69 aA
	BRS Esmeralda	7.2 ± 2.83 aA	4.7 ± 0.51 aA	6.8 ± 2.88 aA
	IRGA 417	5.7 ± 0.76 aA	5.8 ± 1.06 aA	5.2 ± 0.62 aA
	Cultivar	*P* = 0.668	*F*_2,126_ = 0.40	
	Si sources	*P* = 0.985	*F*_2,126_ = 0.01	
	Cultivar × Si sources	*P* = 0.574	*F*_4,126_ = 0.72	
Tl2	Canela de Ferro	3.1 ± 1.40 aA	2.0 ± 0.63 aA	0.4 ± 0.26 aA
	BRS Esmeralda	1.1 ± 0.50 aA	1.3 ± 0.61 aA	0.7 ± 0.3 aA
	IRGA 417	1.8 ± 0.41 aA	2.2 ± 0.62 aA	2.0 ± 0.49 aA
	Cultivar	*P* = 0.171	*F*_2,126_ = 1.80	
	Si sources	*P* = 0.173	*F*_2,126_ = 1.80	
	Cultivar × Si sources	*P* = 0.268	*F*_4,126_ = 1.32	
Tl3a	Canela de Ferro	28.8 ± 5.63 aB	70.2 ± 15.27 aB	184.9 ± 55.01 aA
	BRS Esmeralda	29.6 ± 8.68 aB	50.5 ± 27.20 aA	66.6 ± 38.58 aA
	IRGA 417	90.4 ± 32.27 aA	27.6 ± 6.17 aB	47.9 ± 13.13 aB
	Cultivar	*P* = 0.095	*F*_2,126_ = 2.41	
	Si sources	*P* = 0.041	*F*_2,126_ = 3.32	
	Cultivar × Si sources	*P* = 0.010	*F*_4,126_ = 3.53	
Tl3b	Canela de Ferro	21.3 ± 5.27 aB	62.5 ± 14.57 aB	180.0 ± 55.06 aA
	BRS Esmeralda	21.9 ± 8.41 aB	46.4 ± 27.08 aB	63.0 ± 38.41 aA
	IRGA 417	84.3 ± 32.12 aA	22.1 ± 5.44 aB	42.8 ± 12.32 aB
	Cultivar	*P* = 0.107	*F*_2,126_ = 2.29	
	Si sources	*P* = 0.031	*F*_2,126_ = 3.62	
	Cultivar × Si sources	*P* = 0.008	*F*_4,126_ = 3.65	

Means followed by the same lower-case letter within a column (same variable) and upper-case letter within a row do not differ (LSD test, *P* ~ 0.05). Z + Np, non-probing activities; Tl1, penetration of stylets; Tl2, xylem sap ingestion; Tl3a, cellular maceration; Tl3b, ingestion of macerated tissue.

The durations of waveform events per insect (WDEI) (Table 5) were influenced by rice cultivars and Si sources. This was reflected in xylem ingestion (Tl2) and laceration and maceration of stalk tissue (waveform Tl3a). Thus, *G. spinosa* adults performed longer xylem ingestion events on IRGA 417, while Tl3a events lasted longer on Canela de Ferro and BRS Esmeralda without Si, compared with plants of the same cultivars that received Si fertilization.

**Table 5.** Waveform duration per probing event of *Glyphepomis spinosa* (WDEI—min) on different rice cultivars fertilized with different Si sources

Waveform	Cultivar	WDEI—Waveform duration per event per insect		
		Control	K_2_SiO_3_	CaSiO_3_
Z + Np	Canela de Ferro	140.86 ± 39.23 aA	110.28 ± 21.16 aA	120.95 ± 34.20 aA
	BRS Esmeralda	155.28 ± 36.09 aA	170.44 ± 36.50 aA	184.12 ± 29.41 aA
	IRGA 417	127.34 ± 16.04 aA	217.91 ± 84.19 aA	150.32 ± 14.97 aA
	Cultivar	*P* = 0.303	*F*_2,126_ = 1.20	
	Si sources	*P* = 0.741	*F*_2,126_ = 0.29	
	Cultivar × Si sources	*P* = 0.598	*F*_4,126_ = 0.69	
Tl1	Canela de Ferro	0.97 ± 0.14 aAB	0.34 ± 0.02 aB	1.25 ± 0.34 aA
	BRS Esmeralda	2.56 ± 1.07 aA	0.32 ± 0.02 aB	0.84 ± 0.14 aB
	IRGA 417	0.95 ± 0.21 aAB	0.41 ± 0.09 aB	2.10 ± 1.28 aA
	Cultivar	*P* = 0.693	*F*_2,126_ = 0.36	
	Si sources	*P* = 0.033	*F*_2,126_ = 3.55	
	Cultivar × Si sources	*P* = 0.158	*F*_4,126_ = 1.69	
Tl2	Canela de Ferro	2.47 ± 1.06 bA	2.17 ± 0.63 bA	0.54 ± 0.39 bA
	BRS Esmeralda	4.01 ± 1.62 bA	2.31 ± 1.03 bA	5.79 ± 3.08 abA
	IRGA 417	9.90 ± 4.32 aA	3.41 ± 0.71 aA	6.98 ± 1.92 aA
	Cultivar	*P* = 0.013	*F*_2,126_ = 4.53	
	Si sources	*P* = 0.237	*F*_2,126_ = 1.46	
	Cultivar × Si sources	*P* = 0.405	*F*_4,126_ = 1.01	
Tl3a	Canela de Ferro	10.83 ± 2.59 aA	1.68 ± 0.27 abB	1.22 ± 0.17 abB
	BRS Esmeralda	13.31 ± 3.66 aA	1.23 ± 0.16 bB	1.42 ± 0.21 aB
	IRGA 417	1.00 ± 0.10 bB	1.89 ± 0.51 aA	1.10 ± 0.18 bB
	Cultivar	*P* = 0.004	*F*_2,126_ = 5.88	
	Si sources	*P* < 0.001	*F*_2,126_ = 21.1	
	Cultivar × Si sources	*P* < 0.001	*F*_4,126_ = 6.32	
Tl3b	Canela de Ferro	3.61 ± 1.15 aA	1.38 ± 0.21 aA	1.05 ± 0.23 aA
	BRS Esmeralda	2.31 ± 0.66 aA	1.56 ± 0.32 aA	2.13 ± 1.22 aA
	IRGA 417	0.60 ± 0.13 aA	1.25 ± 0.29 aA	0.82 ± 0.27 aA
	Cultivar	*P* = 0.051	*F*_2,126_ = 3.08	
	Si sources	*P* = 0.207	*F*_2,126_ = 1.60	
	Cultivar × Si sources	*P* = 0.118	*F*_4,126_ = 1.89	

Means followed by the same lower-case letter within a column (same variable) and upper-case letter within a row do not differ (LSD test, *P* ~ 0.05). Z + Np, non-probing activities; Tl1, penetration of stylets; Tl2, xylem sap ingestion; Tl3a, cellular maceration; Tl3b, ingestion of macerated tissue.

The percentage of recording time (PRTP) of *G. spinosa* adults was also influenced by the rice cultivars and Si sources (Table 6). The percentage of time spent in non-probing activities (Z + Np) was highest in IRGA 417 and BRS Esmeralda fertilized with Si in both Si sources. The recording time of xylem ingestion (Tl2) was highest in IRGA 417. In addition, the percentage of time spent on laceration and maceration (Tl3a) was highest in Canela de Ferro and BRS Esmeralda without Si. The same behavior was observed in Tl3b.

**Table 6.** Percentage of recording time of probing by *Glyphepomis spinosa* (PRTP—%) on different rice cultivars fertilized with different Si sources

Waveform	Cultivar	PRTP—Percentage of recording time		
		Control	K_2_SiO_3_	CaSiO_3_
Z + Np	Canela de Ferro	55.60 ± 9.20 bB	79.90 ± 3.90 bA	64.80 ± 8.94 bAB
	BRS Esmeralda	59.49 ± 9.90 bB	85.30 ± 6.90 aA	84.90 ± 6.94 aA
	IRGA 417	86.13 ± 2.57 aB	89.90 ± 1.96 aA	89.40 ± 2.71 aA
	Cultivar	*P* < 0.001	*F*_2,126_ = 8.13	
	Si sources	*P* = 0.004	*F*_2,126_ = 5.88	
	Cultivar × Si sources	*P* = 0.257	*F*_4,126_ = 1.35	
Tl1	Canela de Ferro	0.59 ± 0.16 aA	0.28 ± 0.05 aA	0.99 ± 0.49 aA
	BRS Esmeralda	2.06 ± 1.20 aA	0.15 ± 0.01 aA	0.75 ± 0.46 aA
	IRGA 417	0.55 ± 0.14 aA	0.25 ± 0.05 aA	1.51 ± 1.09 aA
	Cultivar	*P* = 0.745	*F*_2,126_ = 0.29	
	Si sources	*P* = 0.134	*F*_2,126_ = 2.06	
	Cultivar × Si sources	*P* = 0.348	*F*_4,126_ = 1.12	
Tl2	Canela de Ferro	1.33 ± 0.73 abA	0.60 ± 0.22 bA	0.11 ± 0.08 cA
	BRS Esmeralda	0.76 ± 0.31 bA	0.72 ± 0.39 abA	0.64 ± 0.31 bA
	IRGA 417	2.33 ± 0.95 aA	1.07 ± 0.36 aA	1.34 ± 0.36 aA
	Cultivar	*P* = 0.041	*F*_2,126_ = 3.32	
	Si sources	*P* = 0.110	*F*_2,126_ = 2.26	
	Cultivar × Si sources	*P* = 0.662	*F*_4,126_ = 0.60	
Tl3a	Canela de Ferro	32.16 ± 8.58 aA	12.00 ± 2.85 aB	23.08 ± 7.35 aAB
	BRS Esmeralda	30.87 ± 8.23 aA	7.95 ± 4.85 bB	10.97 ± 6.42 abB
	IRGA 417	8.27 ± 2.41 bA	5.87 ± 1.64 bB	5.81 ± 1.82 bB
	Cultivar	*P* = 0.003	*F*_2,126_ = 6.11	
	Si sources	*P* = 0.004	*F*_2,126_ = 5.80	
	Cultivar × Si sources	*P* = 0.294	*F*_4,126_ = 1.12	
Tl3b	Canela de Ferro	8.12 ± 4.12 aA	7.12 ± 1.48 aA	10.95 ± 2.73 aA
	BRS Esmeralda	6.18 ± 2.56 aA	5.84 ± 2.20 aA	2.71 ± 0.61 bA
	IRGA 417	2.68 ± 0.42 bA	2.81 ± 0.59 bA	1.86 ± 0.40 bA
	Cultivar	*P* = 0.001	*F*_2,126_ = 6.90	
	Si sources	*P* = 0.954	*F*_2,126_ = 0.04	
	Cultivar × Si sources	*P* = 0.478	*F*_4,126_ = 0.88	

Means followed by the same lower-case letter within a column (same variable) and upper-case letter within a row do not differ (LSD test, *P* ~ 0.05). Z + Np, non-probing activities; Tl1, penetration of stylets; Tl2, xylem sap ingestion; Tl3a, cellular maceration; Tl3b, ingestion of macerated tissue.

## Discussion

Rice is a silicon (Si) accumulating plant (Yang et al. 2018). Although this element is not essential for plants, studies have shown the importance of Si in mediating resistance to a wide variety of pests in rice (Ma et al. 2004, Massey et al. 2006, França et al. 2018). Our results showed that the probing behavior of *G. spinosa* was influenced in rice plants fertilized with Si.

The time to the first stylet probe (initial probing) was prolonged on BRS Esmeralda and IRGA 417 fertilized with potassium silicate, and on Canela de Ferro and BRS Esmeralda fertilized with calcium silicate. However, IRGA 417 fertilized with calcium silicate produced an unexpected effect; stink bugs began probing sooner compared to the control and potassium silicate treatments. In addition, the adult insects spent more time neither probing nor ingesting but simply walking or standing still (Z and Np waveforms) on IRGA 417 and BRS Esmeralda fertilized with potassium silicate and calcium silicate. *Glyphepomis spinosa* adults also spent less time in cellular laceration and enzymatic maceration of stalk tissues (waveform Tl3a) when feeding on the 3 cultivars fertilized with Si compared to the control (no Si). Thus, using EPG to study probing behavior is very promising in studies of plant resistance and resistance induction in rice plants (Yang et al. 2018, Almeida et al. 2020b, Lucini et al. 2021).

Our EPG results also demonstrated differences in probing behavior among rice cultivars. The time spent in non-probing activities (Z and Np waves) was longer in IRGA 417. The time spent ingesting from xylem sap (waveform Tl2) was shortest in BRS Esmeralda and Canela de Ferro while the shortest time of ingesting macerated tissues (waveform Tl3b) was found in BRS Esmeralda and IRGA 417.

Longer periods spent in non-probing activities (Z and Np waves) and shorter periods in ingestion activities (Tl3a waves) in cultivars fertilized with Si may result from greater stalk hardness and tissue resilience caused by silica accumulation (Wu et al. 2017). Si can also form a mechanical barrier that increases the abrasiveness and rigidity of plant tissues, which hinders stylet penetration (Massey and Hartley 2009, Reynolds et al. 2009) and makes cellular laceration of the stalk tissues more difficult.

In conclusion, the current study showed that fertilizing rice plants with Si alters the ingestion behavior; deterrence was evidenced by longer periods without ingestion, prolonged time to the first stylet probe (initial probing), and less time spent in cellular maceration/laceration. Further studies are needed to: determine the effect of Si-induced resistance against pests that damage other parts of the plant such as the panicle, and the use of EPG as a tool to evaluate resistance inducers in plants.
